# Effect of DMSO on Structural Properties of DMPC and DPPC Liposome Suspensions

**DOI:** 10.3390/jfb15030067

**Published:** 2024-03-10

**Authors:** Luísa M. P. F. Amaral, Maria Rangel, Margarida Bastos

**Affiliations:** 1 REQUIMTE, LAQV, Department of Chemistry and Biochemistry, Faculty of Sciences, University of Porto, R. do Campo Alegre, 4169-007 Porto, Portugal; 2 REQUIMTE, LAQV, Institute of Biomedical Sciences Abel Salazar, ICBAS, University of Porto, Rua Jorge Viterbo Ferreira, 228, 4050-313 Porto, Portugal; mrangel@icbas.up.pt; 3 CIQUP, Institute of Molecular Sciences (IMS), Department of Chemistry and Biochemistry, Faculty of Sciences, University of Porto, 4169-007 Porto, Portugal; mbastos@fc.up.pt

**Keywords:** dimyristoylphosphatidylcholine (DMPC), dipalmitoylphosphatidylcholine (DPPC), dimethyl sulfoxide (DMSO), liposomes, differential scanning calorimetry (DSC), electron paramagnetic resonance (EPR)

## Abstract

The study and characterization of the biophysical properties of membranes and drug–membrane interactions represent a critical step in drug development, as biological membranes act as a barrier that the drug must overcome to reach its active site. Liposomes are widely used in drug delivery to circumvent the poor aqueous solubility of most drugs, improving systemic bioavailability and pharmacokinetics. Further, they can be targeted to deliver to specific disease sites, thus decreasing drug load, and reducing side effects and poor adherence to treatment. To improve drug solubility during liposome preparation, DMSO is the most widely used solvent. This raises concern about the potential effect of DMSO on membranes and leads us to investigate, using DSC and EPR, the influence of DMSO on the behavior of lipid model membranes of DMPC and DPPC. In addition, we tested the influence of DMSO on drug–membrane interaction, using compounds with different hydrophobicity and varying DMSO content, using the same experimental techniques. Overall, it was found that with up to 10% DMSO, changes in the bilayer fluidity or the thermotropic properties of the studied liposomes were not significant, within the experimental uncertainty. For higher concentrations of DMSO, there is a stabilization of both the gel and the rippled gel phases, and increased bilayer fluidity of DMPC and DPPC liposomes leading to an increase in membrane permeability.

## 1. Introduction

The study and characterization of drug–membrane interactions are crucial, as biological membranes act as a barrier that the drug must overcome/bypass to reach its active site. These studies contribute to the understanding of the mechanism of action and toxic effects of drugs already in clinical use, characterizing their pharmacokinetic and pharmacodynamic phases, and more importantly, they are most valuable in the rational design and development of new drugs, as these studies allow the selection of the most interesting molecules (with high pharmacological activity and few side effects), thus reducing time and production costs [1].

Although there are numerous membrane models, liposomes (phospholipid vesicles) are the most widely used models in studies of the biophysical properties of membranes and drug–membrane interactions. The use of simplified membrane models allows the establishment of properties and the control of variables that can affect performance, so that the physicochemical principles and specific interactions with membranes are characterized and interpreted in a controlled way. DMPC and DPPC liposomes are commonly used as model membranes because phosphatidylcholines are major components of most mammalian cell membranes. The simplicity of these model membranes, together with the observed high correlation with effects on real biological membranes justifies their frequent use in biophysical studies [2].

Furthermore, liposomes have been widely used in drug delivery; they improve drug solubility, oral bioavailability, pharmacokinetics, and target delivery to specific disease sites. The success of liposomes as drug delivery vectors has been demonstrated through various liposome-based formulations that have gained commercial approval and are currently in use or undergoing clinical trials. As an example, a considerable number of anti-cancer medicines, including DaunoXome^®^, Depocyt^®^, Myocet, and OnivydeTM have been successfully produced since the first liposome formulation (Doxil^®^) was developed [3]. These nano delivery systems are typically sphere-shaped vesicles containing a phospholipid bilayer and an aqueous core; thus, they have the capacity to encapsulate both hydrophobic and hydrophilic drugs [4]. In terms of liposome drug loading, if the drug is lipid-soluble, it can be dissolved together with the lipid components during film preparation, whereas water-soluble drugs can be dissolved in the used buffer media added to the lipid film to prepare the liposome suspension. Nevertheless, it is often considered desirable that the drug is added in the buffer media, to enable better control of liposome’s drug loading capacity, and it is often necessary to use a co-solvent to improve buffer solubility. DMSO is widely used as a solvent in toxicology and pharmacology studies, in cryopreservation of cells, and as a permeabilization enhancer during topological treatments. DMSO is known to alter membrane permeability, can act as a transport vehicle for topical administration of drugs, and therefore it is regarded as a pharmaceutically active compound. Unfortunately, often the used concentrations are not reported. DMSO is generally accepted as nontoxic below 10% (*v*/*v*) and it is often assumed that the effects of DMSO are negligible [5]. Nevertheless, its extensive use has been somewhat controversial over the past years, due to studies reporting in vivo toxicity at low levels of DMSO [5,6,7]. These results highlight safety concerns when using even low concentrations of DMSO as a solvent for in vivo administration in biological assays. Further, its effect on the phospholipid membrane is often neglected in biological studies. 

The often-desired drug aqueous solubility in liposome drug loading and our concern about the potential effect of DMSO on membranes lead us to investigate the influence of DMSO on the behavior of lipid model membranes (liposomes) of DMPC and DPPC by differential scanning calorimetry (DSC) and electron paramagnetic resonance (EPR) methods. 

In this work, we used 5-doxyl stearic acid (5-DSA) as the spin probe. This spin label consists of a nitroxide radical (doxyl) and a hydrocarbonated chain (stearic acid) which allows its intercalation within the lipids that form the liposomes. Following incorporation, the nitroxide radical locates close to the surface of the lipid bilayer, which allows us to get insight on possible surface structure alterations induced by the presence of DMSO.

Most studies in the literature use MLVs prepared by hydrating the lipid film with the DMSO/water mixture (referred to below as “DMSO added on liposome preparation”), while studies in which the DMSO is added to the already prepared lipid suspension are rare (referred to below as “DMSO added after liposome preparation”). Thus, we carried out experiments with both LUVs and MLVs, using the two types of preparation methods to assess if there were differences in the biophysical properties of the membranes resulting from the preparation method and to the liposome structure (MLVs vs. LUVs). The effect of DMSO in the concentration range 0–40% (*v*/*v*) (0.0018–0.145 mole fraction) on the phase behavior of DMPC and DPPC liposomes was examined using DSC, with regards to three parameters: the phase transition temperatures (pre-transition temperature, *T*_p_, that reflects the gel to ripple transition, clearly seen in MLVs), and main phase transition, *T*_m_, (the gel to liquid-crystalline or ripple to liquid-crystalline transition), the respective calorimetric enthalpy, Δ*H* and the width at half-height, Δ*T*_1/2_. The EPR results, all obtained for the fluid phase of DMPC and DPPC LUVs, provide information about possible changes in fluidity with increasing DMSO content.

Finally, a series of experiments were conducted to assess the impact of DMSO on drug–membrane interaction, using sodium dodecyl sulphate, (SDS) and 1-hexyl-2-methyl-3-hydroxypyridin-4-one as probes (chosen to have different hydrophobicity), and DMSO contents up to 10% (*v*/*v*) (0.0274 mole fraction).

## 2. Materials and Methods

### 2.1. Materials

DMPC, DPPC, and 5-DOXYL Stearic acid ammonium salt were from Avanti Polar Lipids, Alabaster, AL, USA, and used as received. The buffer solutions were prepared from 4-(2-hydroxyethyl)-1-piperazineethanesulfonic acid (HEPES), NaCl and NaN_3_, all purchased from Sigma-Aldrich, Germany. The buffer was 10 mM HEPES, with 150 mM NaCl, 0.02% NaN_3_, pH 7.4. SDS (≥98.5%) and DMSO (anhydrous, ≥99.9%) were purchased from Sigma-Aldrich, Darmstadt, Germany, and used as received. 1-Hexyl-2-methyl-3-hydroxy-4-pyridinone (hexylmpp) was synthesized by the reaction of 3-hydroxy-2-methyl-4-pyrone and hexylamine using the protection method in which the hydrogen atom of the enolic group is replaced by a benzyl group [8]. The compound was recrystallized from methanol/acetone, yielding a pure final product, which purity was verified by gas–liquid chromatography (% purity > 99.8). The experiments were performed on an Agilent 4890D gas chromatograph (Agilent, Santa Clara, CA, USA), equipped with a non-polar capillary HP-5 column (cross-linked, 0.05 diphenyl and 0.95 dimethylpolysiloxane by mass fraction) and a flame ionization detector (FID), using nitrogen as the carrier gas and dimethylformamide as solvent. 

The NMR spectra obtained for the compound was 1H NMR (400 MHz, CDCl_3_) d/ppm: 0.91 (t, 3H, CH_3_); 1.34 (s, 6H, CH_2_CH_2_CH_2_); 1.85 (t, 2H, CH_2_); 2.63 (s, 3H, CH_3_); 4.31 (t, 2H, CH_2_); 7.16 (d, 1H, HAr5); 7.79 (d, 1H, HAr6). 

### 2.2. Preparation of Liposomes

**Scheme 1:**
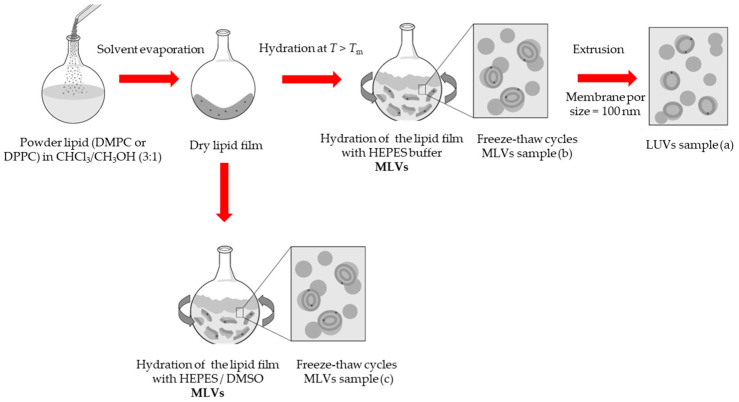
Methodology adopted for liposome samples preparation. (a) LUVs with “DMSO added after preparation”, (b) MLVS with DMSO added to already formed liposomes, and (c) MLVs with “DMSO added on liposome preparation, incorporation method”. In sample (a,b) DMSO is added to the liposome suspensions just prior to the DSC experiment.

The desired amount of DMPC or DPPC powder was dissolved in a mixture of chloroform/methanol (87.4:12.6% (*v*/*v*)), that was subsequently dried under a nitrogen stream to form a lipid film. The traces of organic solvent were removed by keeping the films under vacuum for 16 h. The lipid films were then hydrated for about 1 h with HEPES buffer (10 mM, 150 mM NaCl, 0.02% NaN_3_), pH 7.4 or DMSO/HEPES mixtures, and kept in a water bath at a temperature ~10 °C above the main phase transition of the lipid, with gentle vortex from time to time, thereby creating a multilamellar vesicle’s suspension (MLVs). Thereafter, the MLVs were frozen in liquid nitrogen and thawed above *T*_m_; a cycle that was repeated 6 times. LUVs were prepared from MLVs by the extrusion method under inert (N_2_) atmosphere, using two staked polycarbonate filters with a pore diameter of 100 nm (Whatman, Nucleopore (Malborough, NJ, USA)), in a 10 mL stainless steel extruder from Lipex Biomembranes Inc. (Vancouver, ON, Canada), at a temperature ~10 °C above the gel-fluid transition temperature of each lipid system, performing 15-20 passages through the filter. The sample preparation method is outlined in Scheme 1. The average size of the extruded liposomes was determined by dynamic light scattering analysis (DLS) using a nano Zetasizer from Malvern Instruments (Malvern, UK). The DLS measurements were performed above the transition temperature (*T*_m_), at a total lipid concentration of 0.1 mM, using a He-Ne laser (wavelength 633 nm) as a source of incident light, and operating at a scattering angle of 173°. The prepared samples were monodispersed, with a particle size close to 110 nm, and a polydispersity index (PDI) always < 0.1. For LUVs, the phospholipid concentration was determined after extrusion for each preparation by the phosphomolybdate method [9]. For MLVs, the concentration was obtained by direct calculation from the weighed mass. For all preparations, the final lipid concentration for DSC and EPR experiments was ca. 3 mM. 

### 2.3. Differential Scanning Calorimetry (DSC)

The presence of co-solvents can alter the lipid packing and membrane fluidity, inducing changes in the membrane phase transition temperature, enthalpy of the transition and shape of the DSC peak; all these parameters are retrieved from the DSC curves of liposome preparations. All DSC analyses were performed in a MicroCal VP-DSC microcalorimeter from Malvern (Worcestershire, UK), following the protocol as previously published by our group [10]. In brief, prior to sample loading, blank experiments with buffer (or buffer/DMSO mixtures) in both cells were performed, for subsequent blank correction. The temperature scans were performed at a scanning rate of 60 °C/hour, and three successive heating scans for each sample were always performed, against buffer in the reference cell, to check for reproducibility. The transition temperatures (*T*_m_), the transition enthalpy changes (Δ*H*) and the respective half width at half height (Δ*T*_1/2_) were calculated by integration of the blank corrected heat capacity vs. temperature curve (*C_p_* vs. *T*), using a linear baseline and the Microcal OriginTM software (version 7). Three different sets of preparations were made, with either DMPC or DPPC and different DMSO/HEPES (0% < DMSO < 40%) mixtures, as described below:

(a) Large unilamellar vesicles, LUVs, were prepared in HEPES buffer, and the mixture to be analyzed by DSC was prepared by adding the desired amount of DMSO at room temperature to the LUVs suspension previously prepared in buffer, just prior to the DSC experiment (“DMSO added after preparation”).
(b) Multilamellar vesicles, MLVs, prepared using the same procedure as described above for LUVs (“DMSO added after preparation”).
(c) Multilamellar vesicles, MLVs, prepared by hydration of the lipid film with HEPES buffer already containing DMSO at the desired molar fraction, (“DMSO added on preparation, incorporation method”).

### 2.4. Electron Paramagnetic Resonance (EPR)

**Figure 1:**
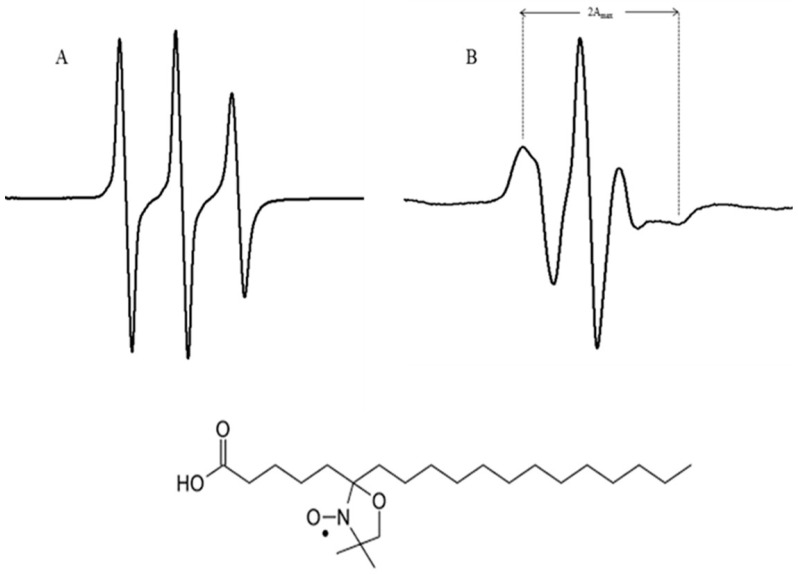
Typical EPR spectra of 5-DSA in: (**A**) in solution, (**B**) DMPC liposome at *T* = 310 K, where 2A_max_ is the maximum hyperfine splitting. The structure of 5-DSA is shown below.

EPR is used to characterize the dynamic behavior of lipids at the molecular level. This spectroscopic approach is very sensitive to changes in the spin–Hamiltonian parameters of the nitroxide spin probes that are commonly used to characterize the structural changes in lipid membrane models. Key features of the EPR spectrum, such as line shape and width, provide insights into the fluidity, phase behavior, and organization of the lipid bilayer [11]. For the EPR studies, we used the spin labelled lipid probe 5-doxylstearic acid (5-DSA), where the nitroxide radical is rather close to the lipids polar head. In the case of the 5-DSA probe in water and DMPC (1,2-dimyristoyl-sn-glycero-3-phosphocholine) lipid bilayers, the EPR spectrum reflects the mobility and arrangement of the spin label. In solution (Figure 1A) the spectrum exhibits a broad, isotropic signal, indicating that the spin label experiences high mobility in a disordered environment. When the probe is introduced into a DMPC bilayer (Figure 1B) the spectrum becomes more structured, reflecting the anisotropic motion of the spin label due to its interactions with the lipid environment.

The spin labelled lipid probe solution was added to the lipids in chloroform/methanol, at a ratio of 1% (mol/mol), and the resulting solution was subsequently dried under a nitrogen stream to form a lipid film. All EPR measurements were made in the fluid phase of each lipid (310 K for DMPC and 323 K for DPPC).

The EPR spectra of 5-DSA incorporated into the lipidic membranes shows characteristics of an anisotropic motion, and the fluidity of the membrane can be estimated from the outermost separation between the spectral extrema, the maximum nitrogen hyperfine splitting (2A_max_), directly obtained by measure in the experimental spectra [12] as shown in Figure 1. The value of 2A_max_ reflects the freedom of rotational motion of the probe close to the polar head groups in the bilayer. This value is known to increase with the decrease in fluidity of the membrane.

Different amounts of DMSO (0–40%) were added to the previously prepared liposome as described in Section 2.3. (a), method “DMSO added after preparation”. The EPR spectra were obtained in triplicate at *T* = 310 K and *T* = 323 K for DMPC and DPPC, respectively. 

The EPR measurements were carried out on a Bruker ELEXSYS E 500 (Bruker, Billerica, MA,, USA equipped with an ER 4222SHQ resonator at Laboratório de Análise Estrutural, Centro de Materiais da Universidade do Porto (CEMUP; Porto, Portugal). The spectrometer was equipped with Bruker N_2_ temperature controller, that maintained the temperature within ±0.1 K during the experiments. 

The spectra were recorded on microwave power 25.18 mW, magnetic field window 150 G (3285 G to 3435 G), modulation amplitude 4 G, conversion time 100 ms using 1024 points, 6 scans.

## 3. Results and Discussion

### 3.1. Effect of DMSO on DMPC and DPPC Liposomes—DSC Results

#### 3.1.1. LUVs, “DMSO Added after Preparation”

**Figure 2:**
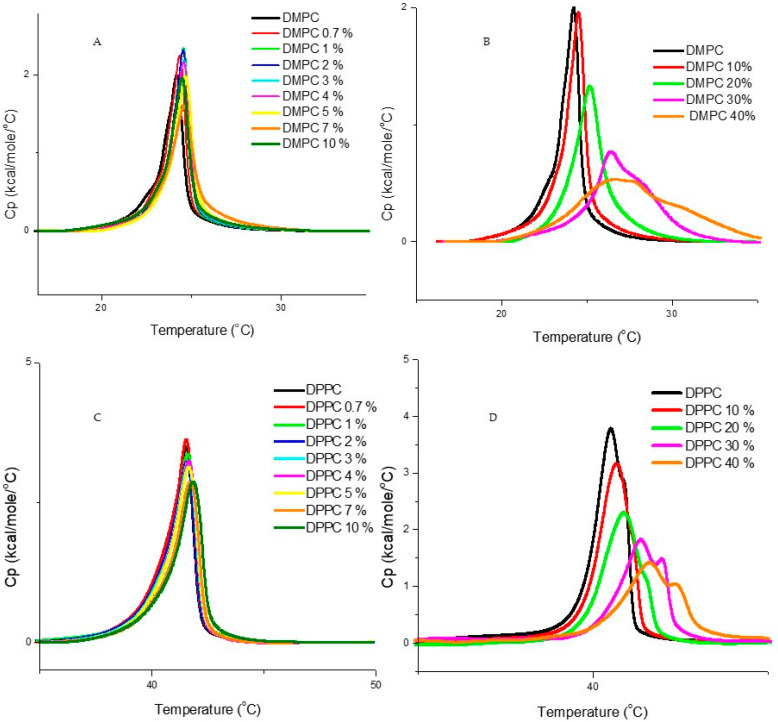
The DSC heating thermograms for DMPC (**A**,**B**) and DPPC (**C**,**D**) unilamellar vesicles (LUVs), with increasing amounts of DMSO, added at room temperature (~22 °C) after liposome preparation, immediately prior to the DSC run (“DMSO added after preparation”). The DMSO content is expressed in the legends, in (*v*/*v*) %. The curves are already corrected for blank (buffer/buffer run), and baseline subtracted.

Figure 2 shows typical heating curves obtained for DMPC and DPPC LUVs containing different molar fractions of DMSO, in the preparation where DMSO was added after liposome preparation, at room temperature (~22 °C), immediately prior to the DSC run. For DMPC liposomes in HEPES buffer, the values obtained for the main-transition temperature (*T*_m_), enthalpy of transition (Δ*H*) and peak half-width (Δ*T*_1/2_) were 24.5 °C, 18.0 kJ·mol^−1^, and 0.8 °C, respectively, whereas for DPPC liposomes in the same conditions the corresponding values were 41.3 °C, 32.9 kJ·mol^−1^ and 1.6 °C; this is in good agreement with the literature [13]. In Table 1, the derived thermodynamic parameters transition temperature (*T*_m_), transition enthalpy change (Δ*H*) and the respective half width at half height (Δ*T*_1/2_), for DMPC and DPPC LUVs at different DMSO contents in the described conditions are displayed. We can see that the addition of DMSO in contents up to 10% (*v*/*v*) does not produce a significant change in the transition behavior of DMPC or DPPC liposome prepared by this method. As DMSO concentration increases from 10% to 40% (*v*/*v*), there is a gradual increase in *T*_m_, a decrease in cooperativity (Δ*T*_1/2_ values increase), a small decrease in transition enthalpy (Δ*H*), and the appearance of a shoulder at higher temperatures, that increases with increase in DMSO contents.

#### 3.1.2. MLVS with “DMSO Added to already Formed Liposomes”

**Figure 3:**
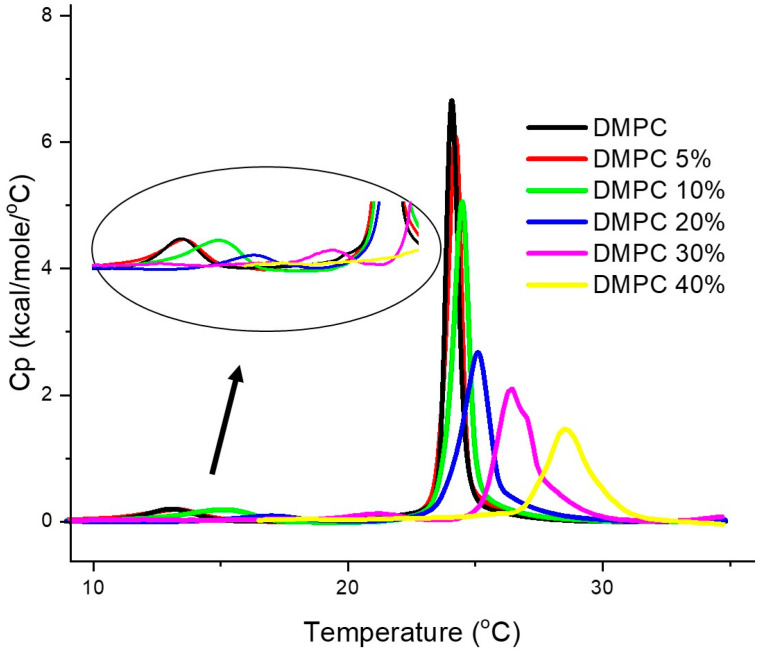
DSC heating thermograms of DMPC multilamellar vesicles, MLVs, “with DMSO added to already formed liposomes” plotted as a function of DMSO content. The DMSO content is expressed in the legends, in (*v*/*v*)%. The curves are already corrected for blank (buffer/buffer run) and baseline subtracted. Insert: enlargement of the pre-transition for the different DMSO contents.

As the effect of DMSO on DMPC and DPPC was the same, in the remaining DSC studies we only used DMPC. The DSC scans in Figure 3 illustrate the overall effect of increasing mole fractions of added DMSO with preparation method (b), i.e., with DMSO added to already formed DMPC MLVs. The retrieved thermodynamic data are listed in Table 2. In addition to the main transition temperature in the absence of DMSO (T_m_ at 23.9 °C), with MLVs we also observe the pretransition, T_p_, the transition between gel and ripple phases. This transition appears clearly for MLVs, and most of the time is not seen in LUVs or is superimposed to the beginning of the main transition, as the two transitions are closer in temperature in the case of LUVs. The peak of the main transition is narrower here, and this is in agreement with the larger cooperativity that is always observed with MLVs, as compared to LUVs. The pre-transition exhibited the expected low Δ*H* values, and *T*_p_ increases significantly with increase in DMSO content, and disappears at 40% DMSO content. Previous studies primarily focused on PC-based bilayers revealed that the effects of DMSO on lipid bilayers depend on its concentration. A study using MLVs prepared from lipid hydration with DMSO/water mixtures, with DMSO in the mole fraction range 0 < X_DMSO_ < 0.18 (where 0.18 mole fraction corresponds to 45% (*v*/*v*)), reports that the temperature of pre- and main transition increase with increasing DMSO mole fraction, reducing the range of temperature over which the ripple phase appears, until eventually there is a direct transition from L_β′_ to L_α_; thus the pre-transition is no longer detected. In contrast, the enthalpy and cooperativity of the transitions were not significantly affected by the presence of DMSO in this concentration range [14]. This shows that DMSO induces a stabilization of both gel and rippled phases, that was interpreted as deriving from dehydration of the polar groups at the bilayer surface that leads to the disappearance of the ripple phase at high DMSO contents [15,16]. Our results by DSC agree with previous findings, using X-ray diffraction [17]. Regarding the main transition, the observed trend and values for T_m_ are similar to the ones obtained for LUVs, with the displacement of *T*_m_ of DMPC to higher temperatures with increasing concentrations of DMSO. The observed decrease in cooperativity, increase in *T*_m_ and decrease in Δ*H* are compatible with a deeper insertion of DMSO here, at the level of the hydrocarbon chains. Thus, overall, the trend observed for the temperatures and enthalpies is similar to what was observed for LUVs with previous preparation method, but it should be noted that the transition enthalpy decreases in this case more significantly as the DMSO content increases.

#### 3.1.3. MLVS with “DMSO Added on Liposome Preparation, Incorporation Method”

**Figure 4:**
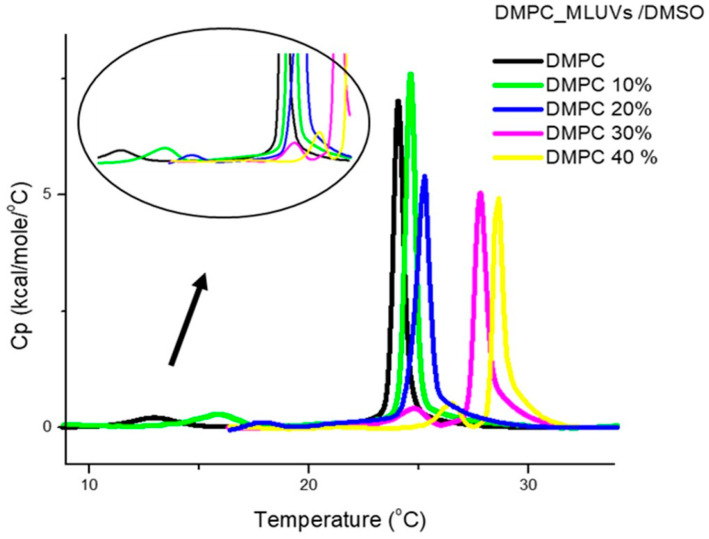
DSC heating thermograms of the DMPC multilamellar vesicles, MLVs, with increasing amounts of DMSO, using “DMSO added on liposome preparation, incorporation method”. Insert: enlargement of the pre-transition for the various DMSO contents. The DMSO content is expressed in the legends, in (*v*/*v*)%. The curves are already corrected for blank (buffer/buffer run) and baseline subtracted.

Figure 4 shows the DSC curves for the DMPC MLVs prepared using a buffer already containing DMSO, for different DMSO/HEPES ratios (0% < DMSO < 40%). The corresponding retrieved data are presented in Table 3. The main effect was a gradual increase in transition and pre-transition temperatures, along with a decrease in Δ*H* values, as in the previous case, where DMSO was added after preparation; interestingly, however, here we saw no change in main transition cooperativity. The observed increase in *T*_p_ per increase in DMSO is larger than the corresponding *T*_m_ increase, leading to a gradual decrease in the temperature gap between the two transition temperatures, until the pretransition disappears for the highest DMSO content (40%). The literature results for MLVs of DPPC showed the same behavior as found here for the DMPC [15].

Globally, the trend of the effect of increasing amounts of DMSO up to 40% is similar, irrespective of preparation method and the use of LUVs or MLVs; namely, DMSO induces initially a stabilization of both gel and rippled phases, until the ripple phase disappears. This is in good agreement with previous reported data [15,16]. There have been conflicting reports and interpretations regarding the partition of DMSO at the water interface. Some data suggest that the DMSO molecules can penetrate the water layer and directly solvate the lipid head group region [16,18,19], while others argue that the dehydration of the head groups occurs due to the stronger preference of DMSO for hydrogen bonding with water over binding to lipid head groups, which results in changes in the water structure near the lipid membrane surfaces [14,15,17,20,21]. In this concentration region, there are also structural reports showing a rapid and almost monotonous decrease of the repeat distance with increasing DMSO concentration for both DPPC and DMPC, up to X_DMSO_ = 0.13 [19,20]. Interestingly, the thickness of the bilayer remains constant for DPPC only up to X_DMSO_ = 0.3, whereas for DMPC it is up to X_DMSO_ = 0.4 [20]. 

In some cases, there is a discrepancy between the MD simulation studies and experimental results regarding the impact of DMSO on membrane properties. At X_DMSO_ < 0.1, molecular dynamics (MD) simulations indicated that due to the presence of DMSO the lipid chains are less packed, with a progressive thinning of the membrane with increasing DMSO content, followed by pore formation and ultimately loss of the membrane integrity [22,23,24]. These findings are contradictory to the experimental results [14,17] that unambiguously showed that the bilayer thickness remains constant and suggest a tighter or more ordered packing of the lipid chains; this is reflected by the increase in the melting temperature. Additionally, membrane rupture at high DMSO concentrations, as predicted by the MD simulations, has never been observed experimentally.

Importantly, our results show an increase in T_m_ in all cases is compatible with a decrease in polar head hydration; this is in agreement with experimental data. Nevertheless, two significant differences should be stressed: (i) the decrease in ΔH values (main transition) as DMSO content increases is more significant for MLVs than for LUVs, irrespective of preparation method (in the case of MLVs); and (ii) when comparing the preparation method for MLVs, another significant difference is observed in the half width at half height; whereas it increases with DMSO content in the case of the first method, when DMSO is added on liposome preparation, it does not change. We interpret the more significant decrease in ΔH values observed for MLVs as resulting from a distribution of the DMSO between the various bilayers being more effective, as the effect has been mainly assigned to a change in hydration. Regarding the observation of a rather constant half width at half height when the second preparation method is used, we suggest that this results from the fact that in the second preparation method the DMSO can be evenly distributed between both leaflets, leading to an effect that could be roughly half the one observed when the first method is used, and we saw that for 20% DMSO the half width at half height is not much increased when the first method is used (see Table 2). 

### 3.2. Effect of DMSO on DMPC and DPPC Liposomes—EPR Results

#### LUVs, “DMSO Added after Preparation”

The EPR study was conducted using DMPC and DPPC LUVs, incorporating the spin probe 5-DSA, where the impact of varying DMSO concentrations (0%, 10%, 20%, 30%, and 40%) was assessed by analysis of the EPR spectra and measurement of the spin–Hamiltonian parameter 2A_max_. EPR data were analyzed using GraphPad Prism (version 8.0.1 for Windows; GraphPad Software Inc., San Diego, CA, USA). The obtained data consisted of three independent experiments.

**Figure 5:**
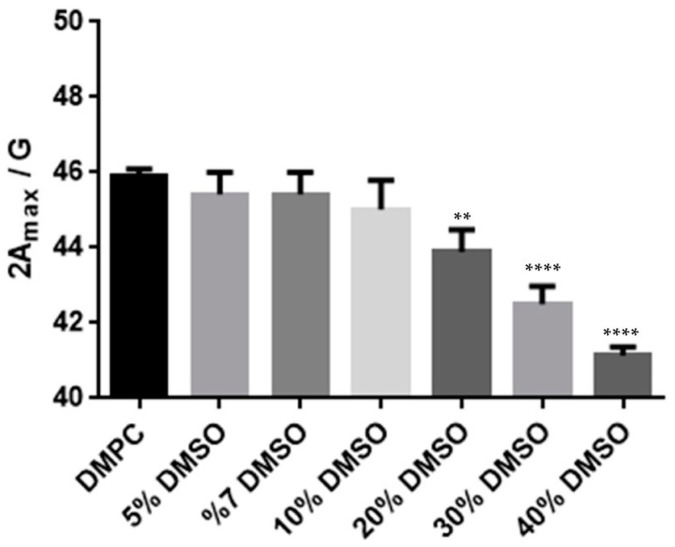
Effect of increasing amounts of DMSO on 5-DSA degree of anisotropy (2A_max_) in DMPC liposomes at T = 310 K (fluid phase, Lα). ** *p* ≤ 0.01; **** *p* ≤ 0.0001.

Figure 5 contain the EPR results obtained at *T* = 310 K for the DMPC liposomes containing the 5-DSA spin labelled, for different DMSO contents. Up to 10% (*v*/*v*) DMSO, no significant change in the 2A_max_ value was observed. However, beyond 20% DMSO, a notable decrease in 2A_max_ was detected; this is indicative of an increased fluidity. 

This finding suggests that DMSO induces a significant change in the local environment of the spin probe, within the fluid phase, particularly evident at higher DMSO concentrations. This aligns with the hypothesis of reduced hydration, wherein DMSO replaces water close to the polar heads. The inability of DMSO to establish hydrogen bonds between the head groups contributes to a less constrained lateral motion of these groups.

**Figure 6:**
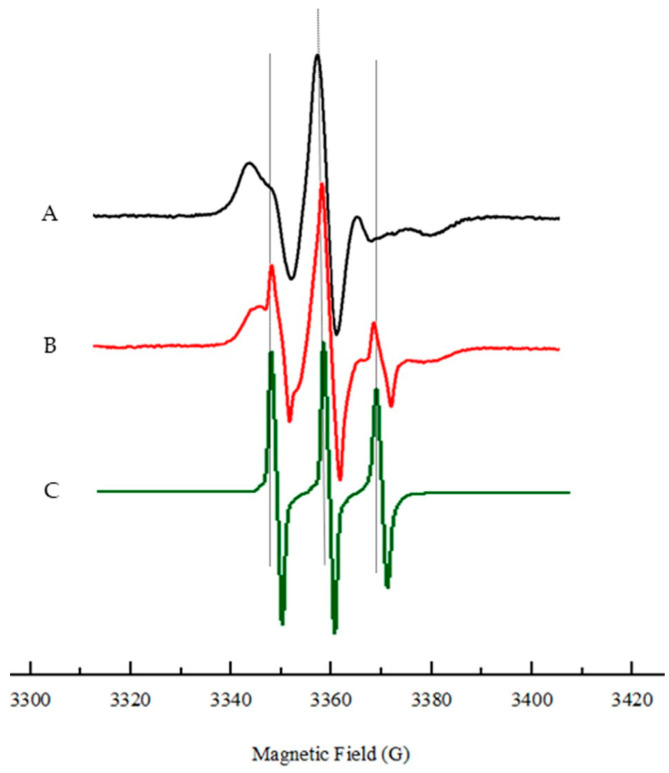
EPR spectrum of spin labelled DPPC liposomes at T = 323 K, with DMSO contents: (A)—0%; (B)—40%; (C)—5-DSA in HEPES/DMSO solution.

On the other hand, in the case of DPPC, a distinct EPR signal attributed to the free 5-DSA spin probe was detected for DMSO concentrations higher than 30%, as depicted in Figure 6. The spectrum on Figure 6-A shows the typical signal of 5-DSA in liposomes suspension in buffer solution, and Figure 6-C in solution (HEPES/DMSO (6:4)). Notably, when we have 40% DMSO, Figure 6-B, the experimental spectrum seems to be the superposition of two signals of the probe: in lipid and in HEPES/DMSO environments. This observation suggests that when DMSO content is higher than 30% the interaction between the spin probe and the DPPC bilayer is disrupted to some extent, leading to the release of the spin probe into the surrounding DMSO rich environment. Indeed, we can also interpret these results as reflecting a deeper DMSO permeation due to preferential solvation of the probe molecules. This effect aligns with the known ability of DMSO to disrupt lipid–lipid interactions, leading to increased bilayer fluidity.

### 3.3. SDS and Hexylmpp Partition to DMPC Liposomes at Different DMSO Contents

When conducting drug–liposome interaction studies using a co-solvent, it is important to carefully interpret the results to enable differentiating between the effects of the drug and the co-solvent. To investigate the influence of DMSO on the thermotropic properties of the liposomes, we studied systems involving liposomes, two different drugs and different DMSO contents. Liposomes were made from DMPC, and we used two drugs with different hydrophobicity: SDS, chosen because its partition to lipid membranes is well characterized [25]; and hexylmpp, because it is a drug with low solubility in water, where DMSO would most likely need to be used to enable aqueous solution studies [26]. The experiments were carried out by adding the drug to the DMPC suspensions, to which different amounts of DMSO up to 10% were added. The hypothesis we wanted to test was how different DMSO contents would affect the lipid phase transition and whether we would get different results when using substances with different hydrophobicity at the same DMSO content.

We used two SDS concentrations, 200 µM and 500 µM, and varied DMSO content between 0–10% (*v*/*v*). The results (Table 4) show that there is no change in the thermodynamic parameters T_m_, ΔH and ΔT_1/2_ up to 5% DMSO, and just a very small, gradual increase in T_m_ is observed at 7 and 10% DMSO. These results are in line with the ones obtained when just DMSO was added (Table 1), indicating that the partition of SDS to the lipid membrane at these low concentrations, 200 µM and 500 µM, is not changing due to the presence of DMSO up to 10%.

The results obtained for hexylmpp are shown in Table 5. In this case, we observe that when increasing the concentration of DMSO a small but significant increase in Tm was observed, especially at the highest DMSO contents 7 and 10%. We interpret this result as reflecting the transfer of hexylmpp from the membrane to the aqueous phase when the DMSO content increases, due to the increase in solubility of hexylmpp in DMSO rich solution, as compared to buffer only (or with low DMSO content) solvent. This can result from a smaller tail in hexylmpp (6 °C) as compared to SDS (12 °C), that is reflected in a lower tendency to partition to lipid membranes.

## 4. Conclusions

In the present work, we have systematically studied the influence of DMSO on the biophysical properties of phospholipid bilayers. In our experiments, we used model membranes composed of phosphatidylcholine, which is a zwitterionic lipid. Two phosphatidylcholines with different acyl chain lengths were used (DMPC and DPPC).

The results obtained from DSC show that upon addition of DMSO up to 40% there is a stabilization of both the gel and rippled gel phases in DMPC and DPPC liposomes, with a modest decrease in the enthalpy change of the main transition. For MLUVs the liposome preparation method demonstrated a distinct impact on liposome cooperativity, suggesting that the distribution of DMSO between leaflets plays a crucial role in liposomal behavior. Our study further revealed that the addition of DMSO up to 10% had minimal impact on the thermotropic properties of phospholipid bilayers.

EPR results indicated a decrease in 2A_max_ in DMPC, suggesting DMSO’s pronounced impact on lipid bilayer packing density and disruption of lipid–lipid interactions. In DPPC, a more complex interplay was observed, possibly due to altered spin probe association and increased solubility in the DMSO-rich environment.

The second set of experiments with SDS and hexylmpp revealed an increase in hexylmpp solubility with rising DMSO concentration. This suggests that DMSO can alter lipid–lipid and drug–lipid interactions within liposomes, influencing drug incorporation, retention, and overall thermodynamic parameters.

Understanding these changes is crucial for gaining insight into the effects of solvents on lipid membrane dynamics, which have implications in drug delivery systems and other applications involving lipid-based formulations. Therefore, it is important to conduct systematic studies to evaluate the thermotropic properties of the liposomes in the presence of both DMSO and the drug of interest, taking in consideration the specific characteristics of the drug, liposomes, and DMSO concentration. This will help to determine if any changes occur and provide a better understanding of the overall behavior of the liposome system in the presence of both DMSO and the drug.

In summary, the interactions between DMSO, liposomes, and drugs can be quite complex and influenced by numerous factors that need to be considered when designing new liposome drug delivery vectors, with or without the aid of co-solvents.

## Figures and Tables

**Table 1:** Main phase transition thermodynamic parameters (*T*_m_, Δ*T*_1*/2*_ and Δ*H*) of DMPC and DPPC LUVs, for increasing amounts of DMSO, added to already formed liposomes ^a^.

X_DMSO_	% DMSO (*v*/*v*)	*T*_m_ /°C	Δ*T*_1/2_/°C	Δ*H* kJ·mol^−1^	*T*_m_/°C	Δ*T*_1/2_/°C	Δ*H* kJ·mol^−1^
		DMPC			DPPC		
0	0	24.5	0.8	18	41.3	1.6	33
0.0018	0.7	24.5	0.8	19	41.4	1.6	33
0.0026	1.0	24.5	0.8	19	41.3	1.7	32
0.0051	2.0	24.6	0.8	19	41.3	1.7	32
0.078	3.0	24.6	0.8	19	41.4	1.7	31
0.010	4.0	24.7	0.9	18	41.4	1.8	31
0.013	5.0	24.7	0.8	18	41.5	1.8	32
0.019	7.0	24.7	0.9	17	41.5	1.8	31
0.027	10	24.8	1.0	17	41.6	1.9	32
0.060	20	25.2	1.7	17	41.6	2.6	30
0.098	30	26.2	3.5	17	42.4	2.9	30
0.14	40	27.9	5.2	17	42.8	3.2	27

^a^ The estimated uncertainty is ±0.2 °C for the transition temperature, ±0.2 °C for the half width at half height, and ±1.5 kJ·mol^−1^ for Δ*H*.

**Table 2:** Phase transition thermodynamic parameters (*T*_p_, Δ*T_1_*_/2,_ Δ*H*_p,_ *T*_m_, Δ*T_1_*_/2_ and Δ*H*) for DMPC MLVs, with DMSO added to already formed liposomes, using DMSO contents between 0–40% (*v*/*v*) ^a^.

X_DMSO_	% DMSO (*v*/*v*)	*T*_p_/°C	Δ*T*_1/2_/°C	Δ*H_p_* kJ·mol^−1^	*T*_m_/°C	Δ*T*_1/2_/°C	Δ*H* kJ·mol^−1^
0	0	13.0	2.2	2	23.9	0.6	21
0.013	5.0	13.2	2.7	3	24.2	0.6	21
0.027	10	15.2	3.3	3	24.4	0.7	20
0.060	20	17.9	3.8	2	25.2	1.2	17
0.098	30	21.5	3.8	2	26.1	1.3	16
0.14	40	---	---	---	28.2	2.0	15

^a^ The estimated uncertainty is ±0.2 °C for the transition temperature, ±0.2 °C for the half width at half height, and ±1.5 kJ·mol^−1^ for Δ*H*.

**Table 3:** Thermodynamic parameters of the phase transition of DMPC MLVs (*T*_p_, Δ*T_1_*_/2,_ Δ*H*_p,_ *T*_m_, Δ*T_1_*_/2_ and Δ*H*), with increasing amounts of DMSO ^a^.

X_DMSO_	% DMSO (*v*/*v*)	*T*_p_/°C	Δ*T*_1/2_/°C	Δ*H_p_* kJ·mol^−1^	*T*_m_ /°C	Δ*T*_1/2_ /°C	Δ*H* kJ·mol^−1^
0	0	13.1	2.3	2	23.9	0.6	21
0.027	10	15.8	2.2	3	24.6	0.6	22
0.060	20	18.2	1.6	2	25.5	0.6	22
0.098	30	24.2	1.3	2	27.5	0.6	18
0.14	40	26.0	1.0	2	28.3	0.6	16

^a^ The estimated uncertainty is ±0.2 °C for the transition temperature, ±0.2 °C for the half width at half height, and ±1.5 kJ·mol^−1^ for Δ*H*.

**Table 4:** Thermodynamic parameters for the main phase transition of DMPC LUVs (*T*_m_, Δ*T*_1/2_ and Δ*H*) using two different SDS concentrations and DMSO contents up to 10% (*v*/*v*) ^a^.

X_DMSO_	%DMSO (*v*/*v*)	*T*_m_/°C	Δ*T*_1/2_/°C	Δ*H* kJ·mol^−1^	*T*_m_/°C	Δ*T*_1/2_/°C	Δ*H* kJ·mol^−1^
		SDS 200 µM			SDS 500 µM		
	DMPC	24.5	1.0	18	24.5	1.0	18
0	0	23.7	1.0	18	23.0	1.0	19
0.0078	3.0	23.7	1.0	18	23.0	1.0	19
0.013	5.0	23.7	1.0	18	23.1	1.0	19
0.019	7.0	23.8	1.0	18	23.2	1.0	18
0.027	10	23.9	1.0	18	23.5	1.0	17

^a^ The estimated uncertainty is ±0.2 °C for the transition temperature, ±0.2 °C for the half width at half height, and ±1.5 kJ·mol^−1^ for Δ*H*.

**Table 5:** Thermodynamic parameters for the main phase transition of DMPC LUVs (*T*_m_, Δ*T*_1/2_ and Δ*H*) with 5 mM hexylmpp and DMSO content up to 10% (*v*/*v*) ^a^.

X_DMSO_	% DMSO (*v*/*v*)	*T*_m_/°C	Δ*T*_1/2_/°C	Δ*H* kJ·mol^−1^
		Hexyl 5 mM		
	DMPC	24.5	0.8	19
0	0	22.6	0.8	21
0.0078	3.0	22.7	0.8	21
0.013	5.0	22.7	0.8	20
0.019	7.0	23.0	0.8	22
0.027	10	23.5	1.0	23

^a^ The estimated uncertainty is ±0.2 °C for the transition temperature, ±0.2 °C for the half width at half height, and ±1.5 kJ·mol^−1^ for Δ*H*.

## Data Availability

Data are contained within the article.
